# Follow-up study of Gambian children with rickets-like bone deformities and elevated plasma FGF23: Possible aetiological factors^☆^^☆☆^

**DOI:** 10.1016/j.bone.2011.10.009

**Published:** 2012-01

**Authors:** Vickie Braithwaite, Landing M.A. Jarjou, Gail R. Goldberg, Helen Jones, John M. Pettifor, Ann Prentice

**Affiliations:** a MRC Human Nutrition Research, Elsie Widdowson Laboratory, Cambridge, UK; b MRC Keneba, The Gambia; c Department of Paediatrics, Faculty of Health Sciences, University of the Witwatersrand and Chris Hani Baragwaneth Hospital, Johannesburg, South Africa

**Keywords:** Calcium, FGF23, Gambia, Kidney, Phosphate, Rickets

## Abstract

We have previously reported on a case-series of children (n = 46) with suspected calcium-deficiency rickets who presented in The Gambia with rickets-like bone deformities. Biochemical analyses discounted vitamin D-deficiency as an aetiological factor but indicated a perturbation of Ca–P metabolism involving low plasma phosphate and high circulating fibroblast growth factor-23 (FGF23) concentrations.

A follow-up study was conducted 5 years after presentation to investigate possible associated factors and characterise recovery. 35 children were investigated at follow-up (RFU). Clinical assessment of bone deformities, overnight fasted 2 h urine and blood samples, 2-day weighed dietary records and 24 h urine collections were obtained. Age- and season-matched data from children from the local community (LC) were used to calculate standard deviation scores (SDS) for RFU children.

None of the RFU children had radiological signs of active rickets. However, over half had residual leg deformities consistent with rickets. Dietary Ca intake (SDS-Ca = − 0.52 (0.98) *p* = 0.04), dietary Ca/P ratio (SDS-Ca/P = − 0.80 (0.82) *p* = 0.0008) and TmP:GFR (SDS-TmP:GFR = − 0.48 (0.81) *p* = 0.04) were significantly lower in RFU children compared with LC children and circulating FGF23 concentration was elevated in 19% of RFU children. Furthermore an inverse relationship was seen between haemoglobin and FGF23 (R^2^ = 25.8, *p* = 0.004).

This study has shown differences in biochemical and dietary profiles between Gambian children with a history of rickets-like bone deformities and children from the local community. This study provided evidence in support of the calcium deficiency hypothesis leading to urinary phosphate wasting and rickets and identified glomerular filtration rate and iron status as possible modulators of FGF23 metabolic pathways.

## Highlights

► Low dietary calcium and eGFR in Gambian children with a history of rickets. ► High FGF23 and urinary phosphate loss in Gambian children with a history of rickets. ► Supports the calcium deficiency hypothesis as the aetiology of Gambian rickets. ► Iron status may be involved in FGF23 metabolic pathways.

## Introduction

There is increasing evidence of the occurrence of nutritional rickets in tropical countries where UVB-containing sunshine is abundant [1]. Studies of children with rickets in South Africa, Nigeria and The Gambia have reported vitamin D status above the range characteristic of vitamin D-deficiency rickets, as measured by plasma concentrations of 25-hydroxyvitamin D (25OHD) [2]. Low dietary calcium has been suggested as a possible explanation of this so-called “sunshine paradox”. Children with rickets in these countries have shown similar blood biochemical profiles with elevated 1,25-dihydroxyvitamin D (1,25(OH)_2_D), parathyroid hormone (PTH) and total alkaline phosphatase (TALP) coupled with low plasma phosphate (P), normal to low plasma calcium (Ca) and a low dietary calcium intake [2–4].

A clinical case-series of 46 children with bone deformities consistent with rickets, conducted in The Gambia, indicated abnormally elevated concentrations of plasma fibroblast growth factor-23 (FGF23) in the majority of cases [2]. The hypothesis presented by Prentice et al. [2] linked a chronically low dietary calcium intake with an elevated plasma FGF23 concentration, resulting in excessive urinary phosphate loss and rickets (Fig. 3). Following treatment with calcium and vitamin D, FGF23 concentrations (as measured with the Immutopics C-terminal FGF23 assay) remained consistently elevated over a 6–12 month period, suggestive of a long-standing, chronic abnormality of phosphate regulation predisposing to rickets.

This follow-up study (RFU) on 35 of the 46 children from the original clinical case-series was conducted 5 years after initial presentation. Results from the RFU children were compared with age- and season-matched data from local community children (LC children).

This study was designed to test whether there were differences in dietary Ca intake, plasma FGF23 concentrations and urinary phosphate excretion between RFU and LC children and to identify other potential contributing pathologies to the aetiology of rickets, such as a perturbed vitamin D metabolism, impaired renal tubular function and poor liver function.

## Materials and methods

### Study approvals

Written informed consent was obtained from parents of the children involved in the study. Ethical approval was given by The Gambian Government/MRC Laboratories Joint Ethics Committee.

### Children at follow-up (RFU children)

The 46 children in the original case-series were those who had attended clinics in MRC Fajara or MRC Keneba, The Gambia, between July 1999 and March 2002 with a presentation of leg deformities consistent with rickets [2]. Most were from the West Kiang province. Attempts were made to trace all these children for recruitment into the follow-up study. 35 children (12 female, 23 male, median (IQR) age 8.5 (2.6 years) were available and were included in RFU. The mean (SD) time interval between presentation and follow-up was 5.3 (0.5) years (range 4.2–6.0 years). All measurements on these children were made during May to September 2006.

### Local community children (LC children)

Age- and season-matched data were obtained from a community study which provided anthropometry, biochemistry, and dietary measurements from 30 Gambian children (LC children). This study was conducted during September and October 2007. The LC children were selected from the West Kiang Demographic Survey Database and were divided into three age bands ranging from 6 to 18 years, with the aim of recruiting a representative sample of 5 girls and 5 boys in each age band. West Kiang was divided into 5 geographical areas and 1 male child and 1 female child were randomly selected from each of the areas in the age bands 6.0–9.9 years (AG1), 10.0–13.9 years (AG2), and 14.0–17.9 (AG3) years. Exclusion criteria included the current use of medication affecting bone mineral metabolism, intestinal, hepatic or renal function, and reported illness in the week preceding the study.

### Characterisation of bone deformity and clinical assessment

A health check was carried out on RFU and LC children, paying particular attention to complaints or signs relating to bone, renal, intestinal and hepatic health. In addition for RFU children, a more detailed clinical assessment was conducted to identify the presence of any clinical signs and symptoms of rickets including seizures, frontal bossing, enlarged costochondral junctions, enlarged wrists or ankles, leg pain, difficulty walking and knock-knee, bow-leg or windswept deformity.

Anteroposterior radiographs and medical photographs were taken of both knees and both wrists of RFU children. Radiographs were scored by a consultant paediatrician (JMP) using a 10-point scoring system developed by Thacher et al. [5]. RFU children were diagnosed as having active rickets, on the basis of a Thacher radiographic score > 1.5 or TALP > 960 U/l or both, as in the original case-series [2]. RFU children were identified as having knock-knee, bow-leg or windswept deformity based on both the clinical examination and visual inspection of medical photographs. In order to investigate a genetic predisposition to rickets, the parent or guardian of RFU children were asked whether or not any other member of their family had signs of rickets-like deformities.

### Anthropometry

Standard anthropometry was conducted including weight, standing height and sitting height. Weight was measured to 0.1 kg using a calibrated electronic scale (model HD-314, Tanita B.V., Hoofddorp, The Netherlands). Height was measured to the nearest mm using a portable stadiometer (Leicester Height Measure, SECA, Hamburg, Germany).

### 2-day weighed dietary record

In order to determine the calcium intake of the children a 2-day weighed dietary assessment was carried out by trained field-workers at the homes of the children. Coding of the dietary records was performed using The Gambian Food Composition Tables [6] and an in-house analysis program adapted for use with Gambian foods was used to calculate nutrient intakes [7]. To consider the likelihood that calcium insufficiency was more prevalent in RFU children a yard stick of 200 mg of calcium a day was taken to represent the average bone calcium accretion rate across childhood [8]. The molar ratio of calcium/phosphorus (Ca/P) was determined using the molecular weight of calcium (40.08 g/mol) and phosphorus (30.97 g/mol). The Ca/P of 1 was used, as convention, to represent the optimal molar ratio of Ca/P in the diet [9]. Children were categorised as having a low dietary Ca/P if they had values < 0.33 [10].

### Fasting blood and 2 h and 24 h urine collections

An overnight-fasted, 2 h urine sample was collected between the hours of 07.00 and 09.00. Urinary dipstick tests (Multistix-SG, Bayer, Newbury, UK) for liver function (presence of bilirubin and urobilinogen) and kidney function (presence of protein, haemoglobin, glucose, and leucocyte esterase) were performed on fresh 2 h urine collections. Acidified (HCl 10 μl/ml, laboratory reagent grade SD 1.18, Fisher Scientific) and non-acidified urine aliquots were stored at − 20 °C and then later transported frozen on dry ice to MRC HNR, Cambridge, UK where they were stored at − 80 °C until analysis.

A fasting, antecubital venous blood sample (5–15 ml according to the age of the child) was collected 1 h after the start of the 2 h urine collection and was transferred to pre-cooled lithium heparin (LiHep) and EDTA-coated tubes. Blood ionised calcium (*i*Ca) and haemoglobin (Hb) were measured in the LiHep sample (ABL77, Radiometer Medical, USA) within 10 min and pH 7.4 corrected values for *i*Ca were used. The remainder of the blood was separated by centrifugation at 4 °C within 45 min and frozen at − 20 °C, and later transported frozen on dry ice to MRC HNR, Cambridge, UK where it was stored at − 80 °C until analysis.

24 h urine collections from the children were supervised by trained field-workers at their homes. Urine was directly voided into acid-washed plastic urine containers and was kept in a cool box with frozen ice-packs until transfer to laboratory refrigerators. At the end of 24 h, all urine provided by each child was pooled, total volume measured and then processed as described for the 2 h urine collection.

### Biochemical analysis

The samples were analysed for markers of vitamin D, calcium and phosphate metabolism, and of renal and hepatic function using commercially available methods according to the manufacturers' instructions. EDTA-plasma was used for the analysis of intact PTH and C-terminal FGF23; LiHep-plasma was used for other analyses. PTH was measured by immunoradiometric assay (DiaSorin Ltd, Wokingham, Berks, UK) and FGF23 was analysed using a 2nd generation C-terminal, two-site enzyme-linked immunosorbant assay (Immutopics Inc., San Clemente, CA). For FGF23 the manufacturer's upper limit of the reference range of 125 RU/ml was used as a cut-off of normality. Plasma 25OHD and 1,25(OH)_2_D were measured by radioimmunoassay (DiaSorin, Stillwater, MN, USA and IDS, Tyne and Wear, UK respectively). For 25OHD, < 25 nmol/l was taken as an indicator of increased risk of vitamin D deficiency rickets [11]. Cyclic AMP (cAMP) was measured using the tetramethylbenzide method (R&D Systems-ELISA). The following colorimetric methods (Koni Analyser 20i, Finland) were used to determine plasma analytes: total calcium (TCa), arsenazo III; P, ammonium molybdate: creatinine (Cr), Jaffe; albumin, bromocresol purple; TALP, p-nitrophenol; magnesium (Mg), xylidyl blue I; cystatin C (Cys C), immunoprecipitation; bilirubin, diazo coupling; and aspartase transaminase (AST), enzymatic.

Acidified urine was used to determine urinary (*u*) *u*Ca, *u*P, *u*Cr, *u*cAMP and *u*Mg employing the same colorimetric methods as for plasma. Standards used in urinary assays were acidified prior to use. Urinary concentrations were expressed in moles per unit time.

Assay accuracy and precision were monitored across the working range of the assays using reference materials provided by external quality assurance schemes (NEQAS, Department of Clinical Biochemistry, Royal Infirmary, Edinburgh, UK: DEQAS, Endocrine/Oncology Laboratory, Charing Cross Hospital, London, UK) or purchased commercially (Roche Human Control, Roche Diagnostic Ltd, Lewes, East Sussex, UK) and kit controls supplied by the manufacturer. In addition, an aliquot of a pooled plasma sample was assayed in each batch to monitor possible drift over time and to provide running quality assurance for analytes where no external reference material was available.

### Statistical analysis and calculations

Statistical analysis including multiple regression, 2-sample Student's t-tests and chi-square tests was performed using DataDesk 6.1.1 (Data Description Inc, Ithaca, NY); p ≤ 0.05 was considered statistically significant. Normally distributed data are presented as mean and 1 SD, positively skewed distribution of data is presented as geometric mean and (− 1 SD, + 1 SD) obtained from the antilog of mean (1 SD) for the logged values. Variables with positively skewed distributions were transformed to natural logarithms before further statistical analysis.

Regression analysis of data from the LC children was used to assess the relationships between age (as a continuous variable) and sex with each variable (anthropometric, biochemical or dietary). Sex was not a significant factor in predicting any of the variables with the exception of creatinine, and therefore was not included in the models presented in this paper. However, 25OHD, iCa, P, FGF23, 1,25(OH)_2_D, PTH, Cys C, Cr and albumin were influenced by age. Age-adjustments were therefore included for these variables. To adjust for age in linear regression, age was added as an independent variable in all models.

Standard deviation scores (SDS) were calculated for all variables to enable age-adjusted comparisons to be made between RFU and LC children. As the data from RFU and LC children were collected at the similar time of year, the SDS were, by definition, adjusted for season. SDS was calculated in the following way: [(value _RFU_ − mean_LC_) / SD_LC_] within the specific age bands as indicated in Local community children (LC children). Group differences between RFU and LC children were determined by 2-sample Student's t-tests using SDS values. This method allowed for the small sample size of LC children in each age band and therefore was a more conservative estimate of the significance of group differences than considering the significance of the deviation of the SDS of RFU children from zero. The sample size of 35 RFU and 30 LC children, meant that the study was able to detect significant group differences in SDS of approximately 0.66 SD (two thirds of a standard deviation) or greater, at p ≤ 0.05 with 80% power.

TCa was corrected for albumin (corr-Ca) by normalising to an albumin concentration of 36 g/l using a correction factor of 0.016 mmol TCa/g albumin. This correction factor was calculated from the slope of the relationship between TCa and albumin in LC children [12].

Urinary excretion and clearance data were corrected for age-appropriate body surface area (BSA_age_). BSA was calculated using the Mosteller formula BSA = √((ht (cm) × wt (kg)) / 3600) m^2^ [13] and then corrected to the age-appropriate mean BSA for each LC AG (AG1: 0.81 (0.12) m^2^, AG2: 1.16 (0.17) m^2^, AG3: 1.38 (0.16) m^2^). As no difference was found between BSA_age_ when calculated with standing height or sitting height, standing height was used for all BSA_age_ adjustments.

Estimated glomerular filtration rate (eGFR ml/min), was derived in four ways from equations which use plasma Cys C and/or plasma Cr as markers.

The Cys C based equations include: 1) Cys C-eGFR = [74.835 / (Cys C(mg/l)^1/0.75^)] ml/min [14] and 2) Counahn–Barret ( C-B-eGFR) = [39.1 [ht (m) / Cr (mg/dl)]^0.516^ × [1.8 / Cys C (mg/l)]^0.294^[30 / urea (mg/dl)]^0.169^ × [1.099]^male^ [ht (m) / 1.4]^0.188^] [15].

The Cr based equations include: 3) Schwarz-eGFR = [k × ht(cm) / Cr (µmol/l)], with k = 0.55 (children, ages 2–12 years and girls ages 13–21 years) and k = 0.70 for boys ages 13–21 years) [16]; and 4) creatinine clearance (C_Cr_) = [(*u*Cr mmol/l × *u*Volume ml/min) / *p*Cr mmol/l].

Renal handling of Ca and P was investigated using urinary excretion expressed both as mmol per unit time (2 h and 24 h for *u*Ca, and *u*P) and as mineral clearance (C_Ca_ and C_P_). C_Ca_ and C_P_ were calculated using the following equation: [(*u*Ca or *u*P mmol/l × urine volume l/h) / (plasma TCa or P mmol/l)] [17]. Tubular maximal reabsorption of phosphate (TmP:GFR) (mmol/l) was determined in the following way: Tubular reabsorption of phosphate (TRP) = 1 − {(*u*P/P) × (Cr/*u*Cr)}, if TRP < 0.86 then TmP:GFR = TRP × P mmol/l, if TRP > 0.86 then TmP:GFR = (0.3 × TRP / {1 − (0.8 × TRP)}) × P mmol/l [18].

## Results

### Children lost to follow-up

Of the 46 subjects in the original study, 11 were lost to follow-up; one had died, 4 had moved away from the region, and 6 were not traceable. There was no significant difference in age, sex or proportion with active rickets at presentation between children in RFU and those lost to follow-up. There was also no significant difference in plasma FGF23, 25OHD, 1,25(OH)_2_D, TCa, P, TALP or PTH at presentation between subjects followed-up in RFU and those who were not (data not shown).

### Characterisation of RFU children

The median age of the 35 RFU children was 8.5 (IQR 2.6) years; 66% were male and 34% female. Nine of the 13 subjects with active rickets in the original study were followed up. There was a trend for RFU children to be heavier than LC children, although not significantly (SDS-weight = 0.41 (0.79) p = 0.07). There was no significant difference in standing height, sitting height or BSA between RFU and LC children (SDS-standing height = − 0.17 (0.81) p = 0.4; SDS-sitting height = − 0.06 (0.7) p = 0.8; SDS-BSA = 0.28 (0.81) p = 0.22). None of the RFU children had active rickets as determined by raised TALP and/or Thacher radiographic scoring. However, 19 (54%) had visible lower limb deformities; 10 (29%) had knock-knees, 8 (23%) had bow-legs and 1 (3%) had windswept deformity. Of those with leg deformities, 4 (11%) had switched from bow-legs to knock-knees since presentation, 1 (3%) experienced pain while walking and 2 (6%) experienced pain while running.

With the exception of two RFU children who were siblings, the parents/guardians of RFU children did not report any other cases of rickets-like bone deformities in their family.

### Dietary intake

Table 1 presents the results from the 2-day dietary assessment. Daily calcium intake was significantly lower in RFU than LC children. The mean calcium intake of RFU children was 188 (124, 283) mg/day compared to 305 (167, 556) mg/day in the LC children. 19 (56%) of the RFU children had calcium intakes of ≤ 200 mg/day compared with 7 (29%) of LC children (χ^2^ = 6.51, p = 0.005). Calcium intake increased with age but was consistently lower in RFU than LC children across the age bands.

There was no obvious difference in dietary habits between the two groups but a possible indication that there was a lower consumption of milk in RFU children. Three (8%) RFU children consumed milk (added to porridge at breakfast) on one (*n = 2)* or both days (*n = 1)* of the dietary assessment compared with six (20%) LC children who consumed milk (added to porridge at breakfast) on one (*n = 2*) or both days (*n = 4*) (difference in number of records: χ^2^ = 4.59, p = 0.02). The mean portion of milk per day (g) was significantly lower in RFU children compared to LC children (56 (67) g and 170 (90) g respectively, p = 0.02). The total mean (g) of milk consumed over two days was significantly lower in RFU children compared to LC children (76 (56) g and 307 (213) g respectively, p = 0.04). RFU children who consumed milk were significantly younger than LC children (9.0 (1.52) and 13.1 (1.7) years respectively, p = 0.02).

LC children in AG2 (10.0–13.9 years) had a higher daily calcium intake compared to AG3 (14.0–18.0 years) due to the fact that 5 of the 6 milk drinkers were in AG2. Daily calcium intake remained significantly lower in RFU than LC children when the milk drinkers in LC AG2 were excluded (SDS-calcium = − 0.56 (1.10) p = 0.04). None of the RFU or LC children had dietary Ca/P ≥ 1.0; the highest was 0.5 and 0.7 mol/mol in RFU and LC children respectively. The molar dietary ratio of Ca/P was significantly lower in RFU children compared with LC children but phosphorus intake was similar in the two groups. RFU children had a greater prevalence of low Ca/P with 77% having a Ca/P < 0.33 compared with 41% of LC children (χ^2^ = 8.52, p = 0.002).

### Biochemical profile

All RFU and LC children had plasma 25OHD concentrations > 25 nmol/l. RFU children had significantly lower Corr-Ca concentrations and tended to have lower iCa and P concentrations compared to LC children (Table 2). The mean group differences between RFU and LC children for FGF23, 1,25OH_2_D and TALP were respectively 0.54 SDS, 0.20 SDS and 0.21 SDS greater in RFU children. Although these differences were below the minimum difference detectable as significant given the sample sizes of the study, this pattern paralleled that seen in the original study of children with rickets (non-active) but was less pronounced.

**Fig. 1:**
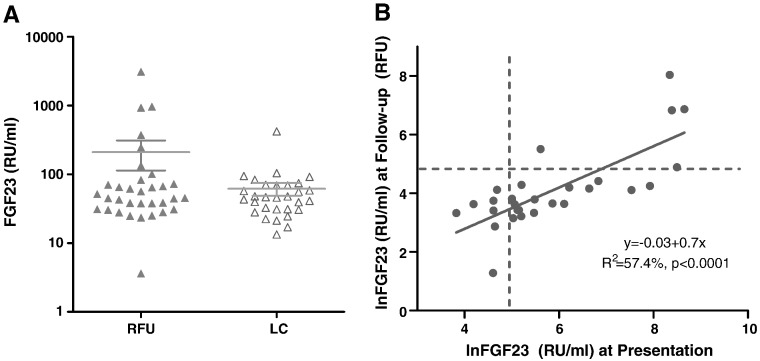
A. FGF23 concentrations of rickets follow-up children (RFU) () and local community children () and mean and SEM. The unadjusted 2-sample Student's *t*-test is p = 0.26. 19% of RFU have [FGF23] > 125 RU/ml, 3% of LC have [FGF23] > 125 RU/ml (χ^2^ = 3.67, p = 0.03). B. lnFGF23 of RFU at follow-up plotted against lnFGF23 at presentation. Broken line indicates the upper-limit assay reference range of 125 RU/ml. The equation of the line is lnFGF23 (RFU) = − 0.3 + 0.7lnFGF23 (presentation), R^2^ = 57.4% (p ≤ 0.0001) (unadjusted for age).

The range of FGF23 concentrations was much wider in RFU children than in LC children due to a pronounced positive skew; 3.5–3091.2 RU/ml and 13.3–421.4 RU/ml respectively (Fig. 1A). Regression analysis indicated a significant correlation between plasma FGF23 at presentation [2] and at follow-up (R^2^ = 56.5%, p ≤ 0.0001) (Fig. 1B). 19% of RFU children (*n = 6*) had FGF23 concentrations > 125 RU/ml compared to 3% of LC children (*n = 1*) (χ^2^ = 3.67, p = 0.03). Although FGF23 concentration decreased from presentation to follow up, children with grossly elevated FGF23 concentrations at presentation remained grossly elevated at follow-up (*n = 3*).

### Liver and kidney function; eGFR and mineral excretion

Urinary dipstick tests for the presence of bilirubin and urobilinogen as markers of liver malfunction were negative for all children in both groups. Plasma concentrations of albumin and AST were significantly elevated and plasma bilirubin significantly lower in RFU than LC children (Table 2).

Urinary dipstick tests for the presence of protein, glucose, Hb and leucocyte esterase as markers of kidney disease or inflammation were negative for all children in both groups.

eGFR as calculated with Cys C based equations (Cys C-eGFR and C-B-eGFR) was significantly lower in RFU than LC children. However, no significant difference was seen in eGFR when using Cr based equations (C_Cr_ or the Schwarz-eGFR) (Table 3).

Mineral handling calculations indicated that TmP:GFR was significantly lower in RFU than LC children and that *u*P excretion over a 24 h period was significantly higher in RFU than LC children. This increase was also reflected in a higher C_P_ over a 24 h period. *u*Ca excretion excretion over 24 h and C_Ca_ were lower in RFU than LC children (Table 3).

### Predictors of FGF23

**Fig. 2:**
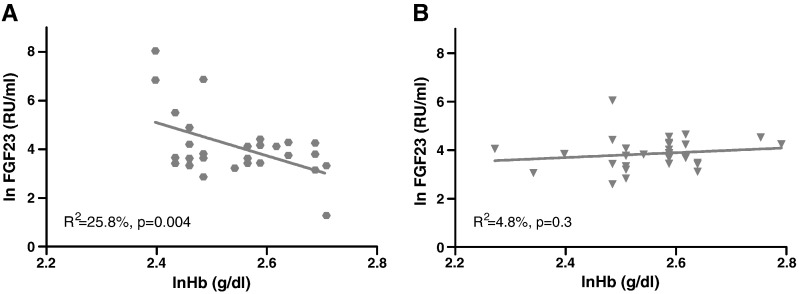
Relationship between lnHb and lnFGF23 in rickets follow-up children (RFU) (A) and local community children (LC) (B). Age adjusted linear regression equations for RFU and LC children are lnFGF23 = 19.2–6.0 lnHb + 0.03 Age, R^2^ = 19.5% (p = 0.01) and lnFGF23 = 0.43 + 1.47 lnHb − 0.03 Age, R^2^ = 4.8% (p = 0.3) respectively. There is a significant lnHb × group interaction (p = 0.003).

**Fig. 3:**
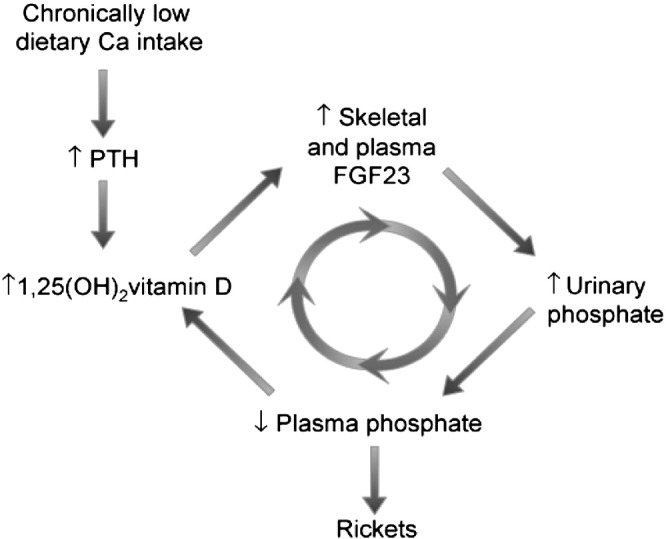
Proposed mechanism relating rickets to urinary phosphate wasting caused by a chronically low calcium intake (reproduced from [2]).

Plasma FGF23 concentration was not correlated with plasma P and 1,25(OH)_2_D or TmP:GFR in either the RFU or LC children. However, Hb concentration was inversely correlated with FGF23 concentration in RFU children (Fig. 2). There was no significant difference in Hb concentration between RFU and LC children (Table 2) but there was a significant Hb × group interaction (*p* = 0.003), indicating a difference in the slope in the relationship between Hb and FGF23 between the two groups.

### RFU children with lasting lower-limb deformities

The median age of the 19 (54%) RFU children with and the 16 (46%) without lasting leg deformities was 8.4 (IQR 2.7) and 8.6 (IQR 2.7) respectively. There was no significant difference in age, standing height, sitting height or weight between RFU children with or without lasting limb deformities. However, those with lasting leg deformities tended to be male (F/M = 4/15) compared to those without lasting leg deformities (F/M = 8/8) (χ^2^ = 3.23, p = 0.04). There was no difference in dietary profile between RFU children with and without lasting limb deformities. Those with leg deformities had significantly higher 1,25(OH)_2_D and significantly lower Cys C-eGFR than those whose deformities had recovered (Table 4). There was no significant difference in Hb concentration (Table 4) or in the relationship between Hb and FGF23 in RFU children with or without leg deformities (data not shown).

## Discussion

The Republic of The Gambia (latitude 13°N) in West Africa has a hot and dry tropical climate with a single wet season from June to October. There is abundant UVB-containing sunshine throughout the year and a lifestyle that does not restrict sunshine exposure but, despite the low prevalence of vitamin D deficiency within the population, there are cases of rickets [2].

The original clinical case series of Gambian children with bone deformities consistent with rickets indicated that 70% of the patients had elevated FGF23 concentrations [2]. This detailed follow-up study conducted 5 years after initial presentation was designed to investigate possible characteristics underlying the aetiology of rickets and long-term abnormalities in bone and mineral metabolism. This study has provided evidence of low calcium intake, persistently elevated FGF23 and increased urinary P excretion. Additionally, this study has not found gross abnormalities suggestive of other potential pathologies contributing to the aetiology of rickets such as a perturbed vitamin D metabolism, renal tubular pathology, or compromised hepatic function. Although there was no evidence from family histories to suggest that these children had a latent genetic disorder of renal phosphate wastage that was unmasked by their low calcium intake, the possibility that they had a genetic predisposition cannot be ruled out.

The rural Gambian diet consists of a high proportion of rice and leaf-based sauces; meat and fish are consumed in relatively small amounts and dairy produce rarely features [19]. The habitual dietary intake of calcium in The Gambia is very low, averaging around 350 mg/day in adults [2], the greatest contributors being from leaves and fruit of the baobab tree [19]. Dietary phosphorus is found in a wide variety of foods and as a result is usually plentiful in a typical Gambian diet [19]. This follow-up study has indicated that, even 5 years post presentation, the RFU children had a significantly lower dietary calcium intake compared to LC children which may be linked to a lower consumption of milk. This may indicate that RFU children may have had an inadequate calcium supply which may have contributed to their poorer skeletal health. Additionally RFU children had a significantly lower calcium-to-phosphorus ratio compared to LC children.

This study also demonstrated that FGF23 concentration has remained above the upper limit of the reference range in 19% of RFU children. Interestingly an elevated FGF23 concentration was also seen in one apparently healthy LC child. Furthermore, urinary phosphate excretion was higher and TmP:GFR was lower in RFU children. Contrary to the original study and despite a greater urinary phosphate excretion in RFU children, circulating P was within the normal range. Furthermore, there was no correlation between FGF23 and P in the RFU children. It is possible that FGF23 no longer regulated P homeostasis in these children either due to a) end-organ resistance to FGF23 or b) a large proportion of inactive C-terminal FGF23 fragments. Alternatively, it is possible that a proportionally greater supply of phosphorus from the gastrointestinal tract enabled the RFU children to maintain normo-phosphataemia in the face of elevated FGF23. This would be explained by a) the higher intestinal P availability and b) an upregulation of the fractional Ca and P absorption due to a low Ca dietary intake [20]. It is thus possible that the absorption of phosphorus was greater in RFU children.

We have identified that eGFR was significantly lower in RFU children compared with LC children when measured by Cys C derived equations. However, when using Cr based eGFR equations no significant differences between the groups were seen. This may be explained by the fact that although both Cys C and Cr are filtered by the glomerulus, a portion of Cr is also secreted by the tubules and excreted in urine [21]. When glomerular filtration is compromised, tubular secretion of Cr is increased in order to maintain normal plasma Cr concentration [22]. The results therefore suggest that RFU children had a less efficient glomerular filtration rate and a compensatory increase in tubular secretion of Cr. An alternative possibility is that the RFU children had a lower lean body mass/weight ratio than LC children resulting in a lower daily release of Cr into the circulation which may have obscured the lower GFR. Cys C is regarded as a more sensitive marker for the calculation of eGFR in children because of problems of interpreting those based on Cr [23]. The data therefore suggest that RFU children had a less efficient GFR but not sufficient to cause clinical problems.

Both the original and follow-up studies measured FGF23 concentrations using the Immutopics C-terminal FGF23 assay which detects both the intact FGF23 hormone and its C-terminal fragments. In the case of RFU it is likely that the elevated FGF23 was reflecting both an increased production of intact and biologically active FGF23 hormone and possibly a greater proportion of presumed inactive C-terminal fragments.

Another intriguing finding was the inverse correlation between Hb and FGF23 in RFU children. As iron deficiency anaemia is endemic in The Gambia [24] a lower Hb in these children is likely to imply a lower iron status. A finding of a relationship between Hb and FGF23 therefore supports previous suggestions of the involvement of iron in FGF23 metabolic pathways [25,26]. Researchers have hypothesised that iron is required for the clearance of FGF23 fragments by the kidney and also that iron may inhibit the cleavage of intact FGF23 [25]. It is possible that the combination of low iron status and lower eGFR may have resulted in greater amounts of circulating FGF23 fragments in RFU children, due to less efficient clearance by the kidney and/or an increased production of C-terminal fragments.

None of the RFU children had radiological signs of active rickets but only half of the children had recovered from their lower-limb deformities. Those with persisting deformities were of similar age but had higher 1,25(OH)_2_D and lower Cys C-eGFR when compared with those who had recovered. A study carried out in Nigeria suggested incomplete distal renal tubular acidosis (idRTA) as a possible cause of differing rates of recovery from rickets-like-deformities [27]. However, it is an unlikely explanation in this Gambian study as idRTA is characteristically accompanied by high *uCa* which was not seen in the RFU children.

In summary the dietary and biochemical profile of the RFU children is in keeping with the calcium deficiency hypothesis; chronically low Ca supply resulting in a 1,25(OH)_2_D-driven increase in FGF23 production, and decreased TmP:GFR and consequent excessive loss of phosphate in the urine. Additionally, this study has suggested the involvement of GFR and iron status in FGF23 metabolic pathways.

## Conflicts of interest

None of the authors has a conflict of interest with respect to the study reported in this paper.

## Figures and Tables

**Table 1:** Dietary assessment.

Dietary intake per day	RFU children *n = 35*			LC children *n = 29*			SDS	
	6.0–9.9 years	10.0–13.9 years	14.0–22.0 years	6.0–9.9 years	10.0–13.9 years	14.0–18.0 years	RFU-SDS	*p*-value
	*n = 29*	*n = 4*	*n = 2*	*n = 9*	*n = 10*	*n = 10*		RFU v LC
Calcium (mg)*	182 (119, 279)	208 (143, 304)	233 (164, 332)	217 (116, 406)	471 (334, 665)	268 (151, 474)	− 0.52 (0.98)	**0.04**
Phosphorus (mg)*	543 (381, 774)	631 (554, 718)	696 (587, 825)	501 (312, 804)	769 (584, 1,013)	647 (404, 1,037)	0.07 (0.75)	0.7
Ca/P (mmol/mmol)	0.27 (0.08)	0.26 (0.09)	0.26 (0.04)	0.34 (0.10)	0.49 (0.15)	0.32 (0.05)	− 0.80 (0.82)	**0.0008**
Energy (kJ)	5560 (1637)	7395 (853)	8105 (442)	5,864 (2179)	7086 (1815)	7473 (2701)	− 0.08 (0.70)	0.7
Protein (g)	12.5 (1.8)	11.1 (1.3)	10.9 (0.1)	36.6 (13.3)	50.7 (10.1)	51.8 (23.1)	− 0.29 (0.88)	0.2
Fibre (g)	27.5 (7.9)	34.6 (3.8)	38.0 (15.2)	31.8 (12.1)	41.9 (11.6)	40.5 (17.2)	− 0.07 (1.31)	0.8
Phytate (g)*	0.72 (0.48, 1.09)	0.84 (0.65, 1.09)	0.73 (0.55, 0.97)	0.72 (0.45, 1.16)	0.98 (0.64, 1.49)	0.89 (0.52, 1.52)	− 0.05 (0.83)	0.8

For normally distributed data, the results are mean (SD); for positively skewed data (denoted by *) the results are geometric mean (− 1SD, + SD). The children are grouped into RFU children (rickets follow-up children) and LC children (local community children) and are then subdivided into age groups. *n* = number of children with data available. RFU-SDS (standard deviation scores) are mean (SD) and are calculated by [value _RFU_ − mean_LC_) / SD_LC_]. *p*-value RFU v LC indicates the 2-sample Student's *t*-test *p*-value (RFU SDS/LC SDS).

**Table 2:** Blood biochemistry.

Fasting blood sample	RFU children *n = 34*			LC children *n = 30*			SDS	
	6.0–9.9 years	10.0–13.9 years	14.0–22.0 years	6.0–9.9 years	10.0–13.9 years	14.0–18.0 years	RFU-SDS	*p*-value
	n = 27	n = 5	n = 2	n = 10	n = 10	n = 10		RFU v LC
25OHD (nmol/l)	61 (14)	57 (12)	67 (12)	61 (17)	52 (18)	46 (16)	0.12 (0.86)	0.6
Corr-Ca (mmol/l)	2.28 (0.08)	2.29 (0.06)	2.27 (0.03)	2.33 (0.04)	2.34 (0.07)	2.34 (0.06)	− 0.97 (1.32)	**0.002**
iCa (mmol/l)	1.13 (0.04)	1.14 (0.02)	1.16 (0.05)	1.14 (0.03)	1.15 (0.03)	1.14 (0.04)	− 0.33 (1.32)	0.2
P (mmol/l)	1.53 (0.13)	1.51 (0.23)	1.06 (0.06)	1.59 (0.20)	1.41 (0.21)	1.45 (0.18)	− 0.27 (0.87)	0.2
FGF23 (RU/ml)*	65 (19, 223)	54 (8, 392)	63 (61, 65)	54 (38, 76)	39 (22, 69)	49 (19, 125)	0.54 (3.33)	0.4
1,25(OH)_2_D (pmol/l)*	246 (192, 315)	323 (181, 578 )	247 (196, 317)	236 (187,298)	265 (197, 356)	287 (224, 368)	0.20 (1.21)	0.5
TALP (U/l)*	299 (243, 369)	332 (273, 405)	133 (132, 133)	280 (220, 356)	264 (204, 342)	252 (190, 335)	0.21 (1.05)	0.4
PTH (pg/ml)*	34.9 (12.7, 96.1)	60.6 (39.7, 92.6)	46.8 (45.8, 47.9)	45.5 (29.4, 70.3)	65.5 (40.5, 105.9)	66.0 (44.2, 98.5)	− 0.55 (2.01)	0.2
Albumin (g/l)	39.9 (2.5)	39.1 (4.5)	39.6 (0.3)	37.8 (2.9)	35.7 (3.5)	36.8 (2.1)	0.78 (0.90)	**0.001**
AST (U/l)*	34.5 (26.4, 45.3)	25.3 (17.7, 36.1)	23.8 (20.1, 28.5)	24.9 (19.9, 30.9)	25.0 (17.9, 34.8)	20.0 (15.6, 25.8)	1.68 (2.06)	**0.0002**
Bilirubin (μmol/l)*	5.3 (3.6, 7.8)	6.8 (5.4, 8.5)	12.7 (4.6, 35.3)	8.2 (6.6, 10.2)	7.6 (5.1, 11.3)	10.8 (6.7, 17.2)	− 1.63 (1.83)	**0.0001**
Cys C (mg/l)*	0.85 (0.75, 0.96)	0.79 (0.71, 0.87)	0.80 (0.69, 0.92)	0.78 (0.70, 0.87)	0.76 (0.65, 0.88)	0.80 (0.69, 0.93)	0.58 (1.04)	**0.02**
Cr (μmol/l)	53.4 (5.1)	56.6 (5.0)	67.9 (4.4)	52.4 (5.0)	59.9 (5.7)	64.0 (8.3)	0.09 (1.00)	0.7
Hb (g/dl)	12.6 (1.16)	13.1 (1.5)	13.5 (0.3)	12.4 (1.3)	12.9 (1.4)	13.5 (1.2)	0.21 (0.88)	0.3
cAMP (pmol/ml)*	38.9 (26.2, 57.9)	48.1 (20.8, 111.0)	31.6 (27.2, 36.8)	61.5 (39.2, 95.6)	68.5 (40.1, 116.9)	60.7 (46.3, 79.6)	− 1.04 (1.02)	**0.0001**
Mg (mmol/l)	0.86 (0.05)	0.78 (0.06)	0.73 (0.03)	0.85 (0.06)	0.84 (0.05)	0.85 (0.03)	− 0.20 (1.34)	0.5

For normally distributed data, the results are mean (SD); for positively skewed data (denoted by *) the results are geometric mean (− 1SD, + SD). The children are grouped into RFU children (rickets follow-up children) and LC children (local community children) and are then subdivided into age groups. *n* = number of children with data available. RFU-SDS (standard deviation scores) are mean (SD) and are calculated by [value _RFU_ − mean_LC_) / SD_LC_]. *p*-value RFU v LC indicates the 2-sample Student's *t*-test *p*-value (RFU SDS/LC SDS).

**Table 3:** eGFR and mineral clearance.

eGFR and mineral clearance	RFU children n = 33			LC children n = 30			SDS	
	6.0–9.9 years	10.0–13.9 years	14.0–22.0 years	6.0–9.9 years	10.0–13.9 years	14.0–18.0 years	RFU-SDS	p-value
	*n = 26*	*n = 5*	*n = 2*	*n = 10*	*n = 10*	*n = 10*		*RFU v LC*
*eGFR*								
Cys C-eGFR	93.7 (16.3)	103.2 (15.0)	101.7 (20)	104.1 (16.1)	109.9 (24.6)	101.3 (20.9)	− 0.55 (0.96)	**0.03**
C-B-eGFR	95.3 (8.1)	100.3 (14.1)	102.7 (8.0)	99.2 (6.4)	110.8 (7.5)	111.5 (12.5)	− 0.74 (1.35)	**0.01**
Schwarz-eGFR	109.9 (9.7)	117.6 (11.7)	120.0 (0.4)	111.1 (6.0)	118.3 (47.4)	138.5 (28.0)	− 0.19 (1.44)	0.5
C_Cr_ (μmol/min)* ^B^	0.53 (0.32, 0.85)	0.56 (0.22, 1.43)	0.69 (0.64, 0.75)	0.46 (0.31, 0.69)	0.54 (0.39, 0.74)	0.70 (3.84, 1.27)	0.27 (1.48)	0.4

*2 h urinary collection*								
P (mmol/2 h) * ^B^	0.68 (0.38, 1.21)	0.54 (0.15, 1.97)	0.68 (0.32, 1.42)	0.48 (0.23, 0.96)	0.46 (0.21, 0.98)	0.54 (0.25, 1.12)	0.44 (0.95)	0.07
C_P_ (mmol/min) ^B^	0.004 (0.003)	0.005 (0.003)	0.006 (0.004)	0.003 (0.002)	0.003 (0.001)	0.003 (0.002)	0.54 (1.33)	0.07
TmP:GFR ^B^	1.60 (0.23)	1.85 (0.33)	1.06 (0.20)	1.88 (0.45)	1.63 (0.33)	1.67 (0.36)	− 0.48 (0.81)	**0.04**
Ca (mmol/2 h)* ^B^	0.03 (0.008, 0.10)	0.02 (0.005, 0.12)	0.15 (0.04, 0.52)	0.02 (0.003, 0.16)	0.04 (0.02, 0.11)	0.06 (0.02, 0.12)	0.07 (0.99)	0.8
C_Ca_ (nmol/min)* ^B^	0.09 (0.02, 0.10)	0.10 (0.04, 0.55)	0.50 (0.13, 1.73)	0.09 (0.02, 0.35)	0.16 (0.06, 0.36)	0.24 (0.14, 0.44)	− 0.03 (1.08)	0.9

*24 h urinary collection*								
P (mmol/24 h)* ^B^	6.2 (3.9, 9.7)	8.9 (6.7, 11.9)	7.4 ^n=1^	4.4 (2.6, 7.5)	8.4 (5.3, 13.4)	11.9 (9.1, 15.6)	0.48 (0.93)	**0.05**
C_P_ (mmol/min) ^B^	0.003 (0.001)	0.004 (0.002)	0.004^n=1^	0.002 (0.001)	0.004 (0.002)	0.006 (0.001)	0.60 (1.05)	**0.02**
Ca (mmol/24 h)* ^B^	0.40 (0.12, 1.35)	0.61 (0.22, 1.69)	1.97^n=1^	0.83 (0.36, 1.89)	0.64 (0.18, 2.20)	0.57 (0.22, 1.43)	− 0.69 (1.44)	**0.03**
C_Ca_ (nmol/min) * ^B^	0.12 (0.03, 0.43)	0.19 (0.04, 0.74)	0.65 (0.18, 2.23)	0.24 (0.10, 0.55)	0.16 (0.04, 0.53)	0.19 (0.09, 0.44)	− 0.55 (1.60)	0.1

For normally distributed data, the results are mean (SD); for positively skewed data (denoted by *) the results are geometric mean (− 1SD, + SD). The children are grouped into RFU children (rickets follow-up children) and LC children (local community children) and are then subdivided into age groups. *n* = number of children with data available. RFU-SDS (standard deviation scores) are mean (SD) and are calculated by [value _RFU_ − mean_LC_) / SD_LC_]. *p*-value RFU v LC indicates 2-sample Student's *t*-test *p*-value (RFU SDS/LC SDS). eGFR (estimated glomerular filtration rate), Cys C (cystatin C), C–B (Counahn–Barret), C_Cr_ (creatinine clearance in mmol/min over 2 h), C_P_ (phosphate clearance in mmol/min — 2 h), TmP:GFR (tubular maximum reabsorption of phosphate in mmol/l), C_Ca_ (calcium clearance in nmol/min — 2 h), C_P_ (phosphate clearance in mmol/min — 24 h) and C_Ca_ (calcium clearance in nmol/min — 24 h). ^B^Indicates variables corrected to an age-appropriate body surface area (BSA_age_) as calculated by mean BSA per LC age band.

**Table 4:** Profile of RFU children with and without lasting leg deformities.

Follow-up	SDS		*p-value*
	Deformity	No deformity	No deformity
	*n = 19*	*n = 16*	v deformity
*Dietary intake per day*			
Calcium (mg)	− 0.48 (0.90)	− 0.51 (1.15)	0.9
Phosphorus (mg)	− 0.01 (0.69)	0.21 (0.83)	0.4
Ca/P (mmol/mmol)	**− 0.80 (0.83)**	**− 0.71 (0.87)**	0.8
Energy (kJ)	− 0.13 (0.75)	0.06 (0.65)	0.4
Protein (g)	0.23 (0.87)	0.42 (0.93)	0.6
Fibre (g)	− 0.27 (1.12)	0.38 (1.50)	0.2
Phytate (g)	− 0.08 (0.84)	0.07 (0.88)	0.6

*Fasting blood sample*			
25OHD (nmol/l)	0.11 (0.85)	0.13 (0.91)	0.9
Corr-Ca (mmol/l)	**− 0.77 (1.13)**	**− 1.18 (1.50)**	0.4
iCa (mmol/l)	− 0.46 (0.99)	− 0.18 (1.63)	0.6
P (mmol/l)	− 0.25 (0.91)	− 0.30 (0.86)	0.9
FGF23 (RU/ml)	1.24 (3.99)	− 0.26 (2.25)	0.2
1,25(OH)_2_D (pmol/l)	0.61 (1.32)	− 0.26 (0.88)	**0.03**
TALP (U/l)	0.30 (0.92)	0.11 (1.20)	0.6
PTH (pg/ml)	0.01 (1.71)	− 1.19 (2.34)	0.1
Albumin (g/l)	**0.79 (1.01)**	**0.77 (0.80)**	0.9
AST (U/l)	**1.94 (1.86)**	**1.38 (2.29)**	0.4
Bilirubin (μmol/l)	**− 2.33 (1.84)**	**− 0.83 (1.50)**	**0.01**
Cys C (mg/l)	**0.96 (0.85)**	0.16 (1.10)	**0.02**
Cr (μmol/l)	0.25 (1.05)	− 0.07 (0.94)	0.3
Hb (g/dl)	− 0.01 (0.73)	0.37 (1.03)	0.2
cAMP (pmol/ml)	**− 1.14 (0.56)**	− **0.93 (1.39)**	0.6
Mg (mmol/l)	0.29 (1.36)	**− 0.76 (1.12)**	**0.03**

*eGFR*			
Cys C eGFR	**− 0.91 (0.72)**	− 0.14 (1.05)	**0.02**
C-B eGFR	**− 1.01 (1.17)**	− 0.44 (1.49)	0.2
C_Cr_ (μmol/min) ^B^	0.32 (1.23)	0.21 (1.74)	0.8

*2 h urinary collection*			
P (mmol/2 h) ^B^	**0.58 (0.71)**	0.30 (1.17)	0.4
C_P_ (mmol/min) ^B^	0.52 (1.02)	0.55 (1.64)	0.9
TmP:GFR^B^	**− 0.58 (0.90)**	− 0.37 (0.70)	0.5
Ca (mmol/2 h) ^B^	0.03 (1.06)	0.12 (0.93)	0.8
C_Ca_ (nmol/min) ^B^	− 0.11 (1.24)	0.06 (0.92)	0.7

*24 h urinary collection*			
P (mmol/24 h) ^B^	0.39 (1.02)	**0.60 (0.82)**	0.5
C_P_ (mmol/min) ^B^	0.53 (1.12)	**0.68 (0.99)**	0.7
Ca (mmol/24 h) ^B^	− 0.63 (1.69)	**− 0.76 (1.09)**	0.8
C_Ca_ (nmol/min) ^B^	− 0.83 (1.92)	− 0.27 (1.19)	0.6

SD-score (as calculated by (value _RFU_ − mean_LC_) / SD_LC_) with LC age bands) and 1 SD of RFU with and without lasting deformity. *p-value* indicates 2-sample Student's *t*-test p-value between RFU with and without deformity. SDS in bold indicates p ≤ 0.05 between RFU group and LC. eGFR (estimated glomerular filtration rate), Cys C (cystatin C), C-B (Counahn–Barret), C_Cr_ (creatinine clearance in mmol/min over 2 h), C_P_ (phosphate clearance in mmol/min — 2 h), TmP:GFR (tubular maximum reabsorption of phosphate in mmol/l), C_Ca_ (calcium clearance in nmol/min — 2 h), C_P_ (phosphate clearance in mmol/min — 24 h) and C_Ca_ (calcium clearance in nmol/min — 24 h). ^B^Indicates variables corrected to an age-appropriate body surface area (BSA_age_) as calculated by mean BSA per LC age band.
